# Mechanical Properties of Glassy Brush Particle Solids Featuring Chain Dispersity

**DOI:** 10.1021/acs.langmuir.6c02933

**Published:** 2026-09-01

**Authors:** Ayesha Abdullah, Hanshu Wu, Yuqi Zhao, Jaejun Lee, Jiajun Yan, Krzysztof Matyjaszewski, Michael R. Bockstaller

**Affiliations:** † Department of Materials Science and Engineering, 6612 Carnegie Mellon University, Pittsburgh, Pennsylvania 15213, United States; ‡ Chemistry Department, Carnegie Mellon University, Pittsburgh, Pennsylvania 15213, United States

## Abstract

The modification of nanoparticles with polymer chains has emerged as a versatile platform to enable hybrid particle building blocks that can be assembled into functional materials with controlled microstructure and enhanced mechanical or physical properties. Often, a combination of high inorganic loading and mechanical strength is desired. Brush chain dispersity is shown to be an effective design parameter to balance Young’s modulus, fracture toughness, and inorganic loading with synthetic versatility. Poly­(methyl methacrylate) brush particle model systems with similar grafting density but varied chain dispersity in the range of 1.1 < *Đ* < 2 were synthesized by varying catalyst concentration in surface-initiated atom transfer radical polymerization (SI-ATRP). Tensile testing revealed an increase of the toughness of brush particle solids with dispersity, whereas the elastic modulus was unaffected. The increased fracture resistance could be attributed to the positively skewed molecular weight distribution of grafted chains, which promoted chain entanglement and craze formation even for low-molecular weight brush chains (i.e., with an average degree of polymerization below the entanglement limit). This renders the “toughening effect” of chain dispersity particularly beneficial for improving the durability of low-molecular brush particle solids (i.e., brush particle solids with high inorganic content) that are susceptible to fracture.

## Graphical abstract

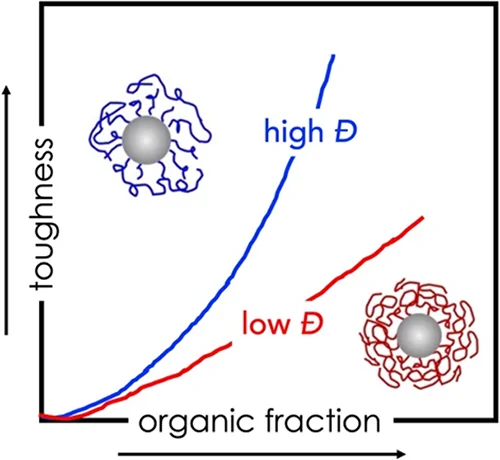

## Introduction

Nanoparticle-based materials exhibit unique properties that arise from quantum-scale confinement effects and the assembled microstructure.^1–5^ These properties are relevant to applications such as molecular semiconductors, hole transport materials, photonic crystals, and photovoltaic cells.^4,6,7^ Deliberate control of nanoparticle interactions has become possible, for example, by modification with surfactants, and has enabled microstructural control through tailoring of assembly conditions.^8–12^ Thus, the assembly of surface-modified nanoparticles has emerged as a viable route to fabricate ordered superlattice structures that mimic diverse metallic and intermetallic lattices.^5,7,13^ However, despite these advances in tailoring the assembly behavior of nanoparticle systems, the processing of assembly structures (in the following called ‘particle solids’) into functional films, e.g., for device integration, remains a challenge. In particular, the fragility of particle solids that arises from short-ranged, hard-sphere interaction potentials dominated by dispersion forces renders particle solids susceptible to crack formation and fracture during processing.^14–17^ Low-molecular weight organic ligands have been shown to enhance cohesive energy density (and hence elastic modulus) of particle solids via the contribution of ligand-dispersion interactions; however, they provide little benefit for the fracture toughness of particle solids.^8^ The latter is a requisite to increase a material’s resistance to modes of failure, such as delamination or crack formation.

The superior toughness of polymers compared to low-molecular weight ligands has inspired the development of surface-initiated polymerization techniques that facilitate the synthesis of polymer-grafted nanoparticles (aka ‘brush particles’, ‘hairy particles’, or ‘nanocomposite tectons’).^18–25^ Polymer modification has been demonstrated to raise the toughness of particle solids through the ability of (flexible) polymers to entangle and raise energy dissipation by inducing microscopic flow processes (e.g., crazing) during crack propagation.^18,26–28^ The realization of brush particles with precisely controlled architecture has been enabled by recent advances in grafting-from polymerization methods, including surface-initiated controlled radical polymerizations (CRP), that provide fine control of the chain length, molecular weight distribution, composition, and density of grafts.^26–35^ The resulting brush particles were demonstrated to present a platform for functional composite materials and a model system to establish structure–property relationships in particle brush solids.

For example, Lee et al.^34^ applied surface-initiated atom transfer radical polymerization (SI-ATRP) to synthesize a library of poly­(methyl methacrylate) (PMMA)- and polystyrene (PS)-grafted silica-based brush particles (core radius: *r*_P_ = 7.9 ± 2.2 nm) with systematically varied grafting density and degree of polymerization. Analysis of the microstructure and mechanical properties revealed that ‘toughening’ of the particle solid is contingent on sufficient length and random walk conformation of polymer tethers to enable entanglement and subsequent craze formation in the solid state; this was later corroborated by molecular dynamics studies.^35–38^ The constraint on length and conformation of polymer tethers implies an inverse correlation of inorganic content and toughness. For some systems, an upper bound of 6% of inorganic volume fraction was reported to realize mechanical properties like the respective linear chain analog. This presents a technological challenge to the utilization of brush particle materials since relevant properties (such as dielectric constant or conductivity) generally positively correlate with inorganic content. Yan et al.^39^ presented a ‘bimodal ligand’ approach via selective deactivation of chain ends during the polymerization process to realize tough brush particle solids with a high (>30 vol %) inorganic fraction. A majority of ‘short’ (low molecular weight) chains effectively prevented aggregation during assembly, while a small fraction of ‘long’ (high molecular weight) chains participated in entanglement and induced toughening of the material. However, despite its elegance, the applicability of bimodal brush architectures is limited due to the complexity of the multistep synthesis process that reduces scalability.

An intriguing alternative strategy to realize processable brush particle solids with increased inorganic content could be the design of ‘disperse’ molecular weight distributions (MWDs) of grafted chains. Synthetic polymers often feature MWDs that are positively skewed (e.g., Schulz–Zimm-type), featuring a high-molecular weight tail that mimics the presence of a small fraction of high-molecular weight species in bimodal distributions.^40^ However, in contrast to bimodal distributions, disperse MWDs can be realized by relaxing control over the radical polymerization process, which benefits both the viability and scalability of the synthetic method. The purpose of the present study was to elucidate the role of chain dispersity in the structures, thermomechanical properties, and deformation behavior of glassy brush particle solids. Activators regenerated by electron transfer (ARGET) SI-ATRP was used to synthesize a library of PMMA-tethered silica brush particle materials with a select variation of the degree of polymerization and dispersity of the PMMA chains.^41,42^ Asymmetry of the MWD was found to promote chain entanglement, craze formation, and energy dissipation in disperse brush systems. Thus, the deliberate introduction of dispersity could provide a viable path toward robust and processable brush particle solids with increased inorganic content. The results also provide context for the interpretation of unexpected property features of disperse brush materials, such as accelerated self-healing, that have been reported recently.^43^

## Materials and Methods

### Materials

Methyl methacrylate (MMA, Aldrich, 99%) was filtered through a basic alumina column to remove the inhibitor. Silica nanoparticles dispersed in MIBK (MIBK-ST) with an average radius of *r*_P_ = 7.9 ± 2.2 nm were donated by Nissan Chemical. Copper­(II) bromide (CuBr_2_, Aldrich, 99%), anisole (Aldrich, 99%), dimethylformamide (DMF, Fisher, 99%), tin­(II) 2-ethylhexanoate (Sn­(EH)_2_, 95%, Aldrich), tetrahydrofuran (THF, VWR 99%), and tris­(1-(methylamino)­ethyl)­amine (ME_6_TREN, Alfa, 99%) were used as received.

### Synthesis of Brush Particles

Synthesis of the 3-(chlorodimethylsilyl)­propyl 2-bromoisobutyrate initiator and surface modification of silica nanoparticles were performed according to established procedures.^30^ The initiator-modified silica nanoparticles, MMA monomer, anisole, DMF, CuBr_2_, and Me_6_TREN were mixed in a sealed Schlenk flask, while a solution of Sn­(EH)_2_ in anisole was prepared. Both solutions were nitrogen-purged. The Sn­(EH)_2_ solution was injected into the Schlenk flask to activate the catalyst complex, and the flask was immediately placed in a 60 °C oil bath. The monomer conversion and polymer molecular weights were monitored using NMR and SEC. Systematically, high-dispersity PMMA-SiO_2_ particles were achieved by tuning [CuBr_2_] according to published procedures^44^ at concentrations less than 10 ppm, they provided polymers with high dispersity, defined as *Đ* > 1.5.

### Nuclear Magnetic Resonance Spectroscopy (NMR)

Monomer conversion was monitored by ^1^H NMR on a Bruker Advance 500 MHz NMR instrument in CDCl_3_ at room temperature.

### Size Exclusion Chromatography (SEC)

Polymer chains were chemically cleaved from the surface of nanoparticles for SEC using previously established procedures.^30^ Polymer number-average *M*_n_ and weight-average *M*_w_ molecular weights were determined using a Waters 515 pump and Waters 410 differential refractometer through PSS columns containing Styrogel at 10^5^, 10^3^, and 10^2^ Å. THF was used as an eluent at 35 °C with a flow rate of 1 mL/min. Linear PMMA was used as the calibration standard. The skewness *U*_3_ and the asymmetry coefficient *A*_s_ = *U*_3_/*s* ^3^, where *s* is the standard deviation of the distribution, were determined from number-weighted SEC traces.

### Thermogravimetric Analysis (TGA)

A TA Instruments 2950 was used to measure the inorganic (SiO_2_) fraction with four steps: (1) jump to 120 °C; (2) hold at 120 °C for 10 min; (3) ramp up at 20 °C/min to 800 °C; and (4) hold for 2 min. TGA plots were normalized to total weight after holding at 120 °C and analyzed using TA Universal Analysis software.

### Transmission Electron Microscopy (TEM)

Monolayer films were cast by using a micropipette to drop-cast 8–10 μL drops of 1–2 mg/mL brush particle solution in toluene onto a 300-mesh carbon-coated copper grid. Nearly monolayer films were spin-coated onto the poly­(acrylic acid) (PAA) substrate, and water immersion lift-off was used to transfer the multilayer film onto a 300-mesh carbon-coated copper grid. Cracks were introduced by bending each grid on a curvature of radius *r* = 0.5 cm. All films were annealed at 120 °C under vacuum for 24 h. The particle microstructure and craze formation were imaged using a FEI Tecnai F20 electron microscope operated at 200 kV. Images were taken on a Gatan Rio high-resolution camera. Micrographs were filtered and binarized, and Voronoi cell analysis was performed in MATLAB using the image processing toolbox.

### Bulk Film Preparation

Brush particles were dissolved in THF solution (∼40 mg/mL) and cast layer-by-layer into a PTFE mold (*L* × *W* × *H* = 15 mm × 5 mm × 0.3 mm) for thick specimens. Solvent evaporation was slowed with a semiclosed system and vapor-supplied atmosphere between each layer. Films were dried in air overnight and annealed at 115 °C for 24 h under light vacuum of 10–15 inHg. Linear PMMA (*N* = 350) samples were cast with the same procedure from 60 mg/mL solution in toluene and annealed at 85 °C for 72 h.

### Tensile Testing

Bulk film specimens were characterized with a TA Instruments RSA-G2 dynamic mechanical analyzer. Samples were strained in tension at a constant linear rate of 0.01 mm/s from a strain-free state. Data were analyzed in MATLAB. Young’s modulus was calculated using the maximum slope of the stress–strain curve at strains below 0.003 mm/mm (confirmed with negligible hysteresis). Toughness was calculated using trapezoidal integration of area under the curve until the fracture point.

### Differential Scanning Calorimetry (DSC)

DSC was performed using a TA Instruments DSC 2500 to observe glass transition characteristics. Samples were held at 120 °C for 180 min to erase thermal history, and then, three cycles across a temperature range of 25 °C < *T* < 170 °C were run at heating/cooling rates of 10 °C/min. Glass transition temperature (*T*_g_) was determined by the inflection point, and glass transition breadth was determined by intersections of tangents that marked onset and offset points.

## Results and Discussion

A series of PMMA-SiO_2_ brush particle samples with systematically varied degree of polymerization *N* and dispersity index (*Đ* = *M*_w_/*M*_n_) were synthesized via ARGET SI-ATRP.^44,45^ The dispersity of the MWD was controlled through concentration of the Cu­(II) catalyst as shown in Figure 1.

**Figure 1:**
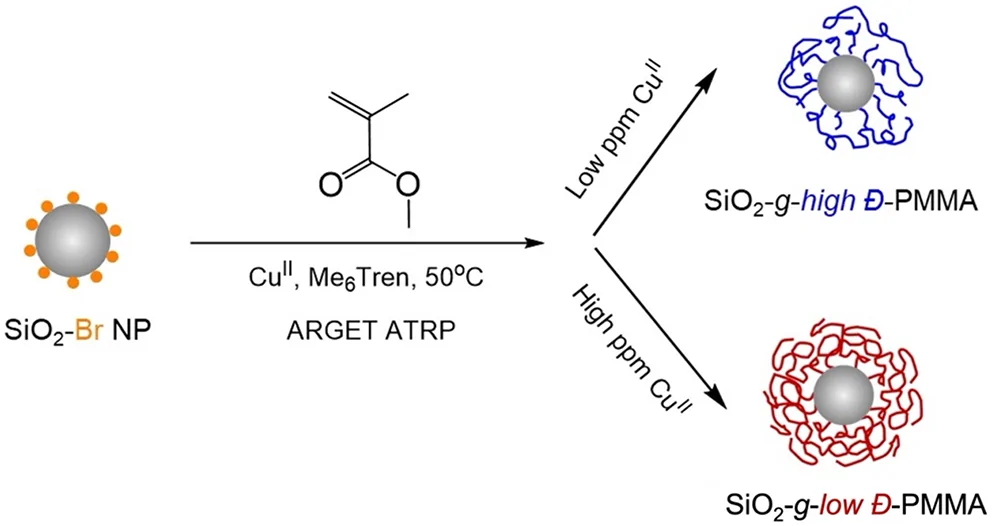
ARGET SI-ATRP of the PMMA brush layer on silica nanoparticles functionalized with the 3-(chlorodimethylsilyl)­propyl 2-bromoisobutyrate initiator. Dispersity of the MWD was controlled by systematic variation of CuBr_2_ catalyst concentration following previously published procedures.^44^

The MWD was determined using size exclusion chromatography (SEC) on chains cleaved from the particles using hydrofluoric acid etching. To selectively determine the impact of chain dispersity, the synthetic process targeted a similar grafting density σ with 0.1 < σ < 0.3 chains/nm^2^ (i.e., all systems are considered to be in the intermediate grafting regime). Brush particles featuring *Đ* < 1.5 were classified as low dispersity. In the following, systems with narrow and high-dispersity brush architecture are identified as “LX” and “HX”, respectively, where “X” represents the sample number. Low-dispersity linear PMMA (*N* = 350) was used as a reference material for mechanical characterization. The molecular characteristics of all brush particle systems synthesized and used in this work are displayed in Table 1.

**Table 1:** Molecular Characteristics of PMMA-Grafted Silica Nanoparticles^a^

sample ID	ϕ_PMMA_	*N*	*M*_n_ (g/mol)	*M*_w_ (g/mol)	σ (nm^–2^)	*Đ*	*A*_S_	*T*_g_ (°C)	*T*_g, breadth_
linear PMMA	1.00	350	35,000	38,500		1.10		89.6	24.6
N1	0.76	526	52,550	62,535	0.11	1.19	1.50		
N2	0.91	526	52,570	65,187	0.35	1.24	0.42	96.0	27.8
N3	0.77	367	36,690	45,863	0.16	1.25	0.34		
N4	0.86	313	31,300	39,125	0.35	1.25	0.30	90.7	28.8
N5	0.91	957	95,690	124,397	0.18	1.30	0.58	95.8	29.2
N6	0.88	642	64,200	83,460	0.20	1.30	0.33	92.0	28.2
N7	0.87	490	49,000	67,700	0.25	1.38	1.16		
H1	0.90	551	55,070	87,740	0.30	1.59	1.94	85.3	30.4
H2	0.90	389	38,910	62,180	0.39	1.60	1.43		
H3	0.74	383	38,320	61,695	0.13	1.61	1.36		
H4	0.81	327	32,650	53,250	0.23	1.63	1.82	83.9	34.0
H5	0.71	228	22,790	38,300	0.19	1.68	1.87	87.6	32.7
H6	0.86	453	45,290	76,087	0.24	1.68	1.67		
H7	0.82	317	31,680	55,730	0.26	1.76	1.89	79.2	35.9
H8	0.74	323	32,260	61,190	0.16	1.90	2.26		
H9	0.77	605	60,480	120,355	0.10	1.99	2.25		
H10	0.87	484	48,400	104,400	0.25	2.04	5.20	79.5	34.2

a Variable definitions: ϕ_PMMA_ is the organic volume fraction of the material; *N* is the degree of polymerization of the polymer; *M*_n_ is the number-average molecular weight; *M*_w_ is the weight-average molecular weight; σ is the grafting density; *Đ* is the dispersity index; and *A*_s_ is the asymmetry coefficient of the molecular weight distribution (see text for more details).^40^ Glass transition temperature *T*_g_ and breadth of transition were determined by DSC.

Thick/thin (monolayer) films were fabricated by mold casting/spin coating from 3%-THF solution and subsequent vacuum annealing at *T* = 120 °C for 24 h. The microstructure of monolayer films was evaluated using TEM imaging. Voronoi cell analysis was used to establish the full width at half maximum (fwhm) of the cell area (*A*) that was used as a metric for film uniformity. Figure 2 depicts the electron micrographs of two representative brush particle monolayers with similar composition (i.e., ϕ_PMMA_ ∼ 0.9) but distinct chain dispersity *Đ* = 1.30 (L5, Figure 2a) and *Đ* = 1.59 (H1, Figure 2b). The micrographs revealed similar fwhm of both materials, thus confirming that brush dispersity did not significantly detriment the structural order of brush particle films.

**Figure 2:**
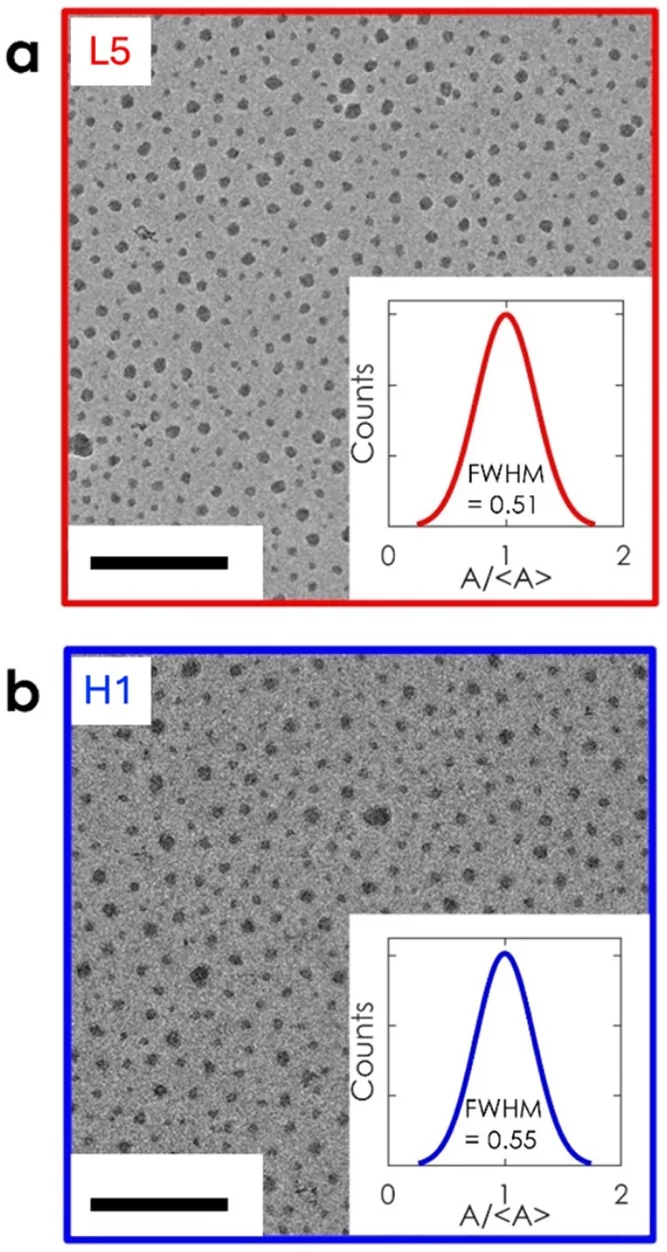
Bright-field TEM micrographs of (a) L5 (ϕ_PMMA_ = 0.91) and (b) H1 (ϕ_PMMA_ = 0.90) with the corresponding normalized Voronoi cell area distributions in the inset. Similar fwhm indicates similar uniformity of L5 and H1. ⟨*A*⟩ = 1404 nm^2^ (L5) and ⟨*A*⟩ = 1543 nm^2^ (H1). Scale bars are 200 nm.

Interestingly, the average Voronoi cell area size ⟨*A*⟩ at a given organic volume fraction was found to be consistently larger for high-dispersity brush architectures, as shown in Figure 3. This increase is unlikely to be related to the somewhat larger grafting density of system H1 but rather could be indicative of larger fractional free volume in disperse brush canopies. The result corroborates simulations by Dodd and Jayaraman^46^ who reported brush height to increase with dispersity due to a reduction in packing frustration (all other parameters considered equal). To further support a positive correlation of brush dispersity with free volume, the glass transition temperatures (*T*_g_) of all systems were determined by DSC. Indeed, the values summarized in Table 1 as well as Figure S1 confirm a minor reduction of *T*_g_ along with an increased width of the glass transition with increasing dispersity index, consistent with an increase in the brush free volume. These results also confirm prior reports on the impact of dispersity on the thermomechanical properties of brush particle materials.^47^

**Figure 3:**
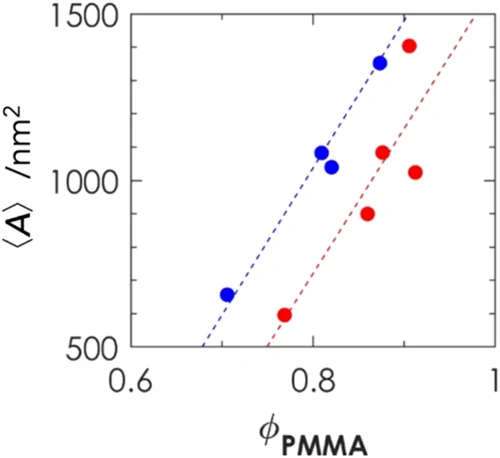
Voronoi cell area average ⟨*A*⟩ of disperse (blue) and low-dispersity (red) SiO_2_–PMMA brush particles as a function of ϕ_PMMA_. At equal polymer volume fraction, the average Voronoi cell area ⟨*A*⟩ of disperse brush systems exceeds that of narrow disperse analogs. The linear fits are provided to guide the eye.

Young’s modulus and toughness were characterized by tensile testing of thick free-standing films (thickness >200 μm). Representative stress–strain curves of high- and narrow dispersity brush particle systems are displayed in Figure 4. Young’s modulus was calculated from the slope of the stress–strain curves at strains ε < 0.003 in the linear elastic regime. Elastic deformation was confirmed by negligible hysteresis within this strain limit (Figure S6). The toughness, i.e., energy per unit volume absorbed before fracture, was calculated by integrating the area under the stress–strain curve until the fracture point. Figure 4 reveals a distinctly different effect of dispersity on Young’s modulus (*E*) and toughness (*U*) of brush particle solids. As exemplified for brush particle solids with a near-equal polymer fraction (L5 and H1), the elastic modulus *E* was independent of dispersity (*E*_L5_ = 1.13 GPa, *E*_H1_ = 1.14 GPa). In contrast, the toughness significantly increased with the dispersity of tethered chains (*U*_L5_ = 0.18 MPa, *U*_H1_ = 0.26 MPa). This is consistent with fracture being dependent on the molecular weight distribution (via entanglement formation).

**Figure 4:**
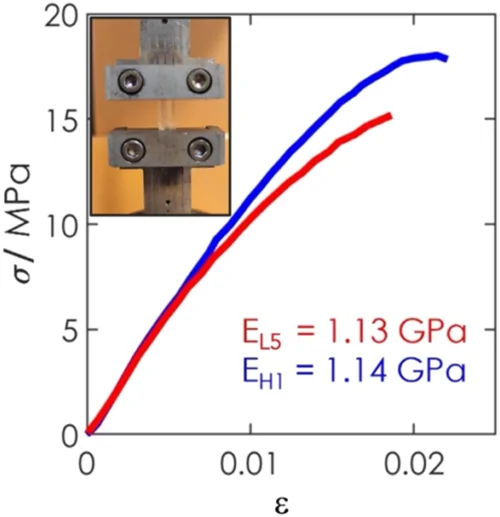
Representative stress–strain curves of narrow (L5, red curve) vs broad (H1, blue curve) dispersity materials measured at a strain rate of 0.01 mm/s along with their respective Young’s moduli (determined in the range ε < 3 × 10^–3^). Analysis of the stress–strain curves yields *E*_L5_ = 1.13 GPa, *E*_H1_ = 1.14 GPa, *U*_L5_ = 0.18 MPa, and *U*_H1_ = 0.26 MPa for the elastic modulus and toughness, respectively. The inset shows the picture of sample H1 during deformation.

The opposing trends were attributed to the different relevant length scales of structure contributing to each property characteristic. Previous studies have shown the elastic modulus of brush particle solids to be determined by polymer dispersion interactions between adjacent brush particles.^8,34,47,48^ Since polymer dispersion interactions are determined by electronic correlation volumes that extend only few repeat units, the effect of chain length is expected to diminish at a moderate degree of polymerization.^49^ Young’s modulus thus rather depends on the chemical makeup of the repeat units compared to the actual length of chains. In contrast, the toughness of amorphous polymers was shown to depend on the formation of chain entanglements (at *N* > *N*_e_) that give rise to energy dissipation through craze formation.^50^ Accordingly, in brush particle solids, toughening through craze formation was expected to be contingent on the sufficient interdigitation between brush layers of adjacent particles such that *N*_i_ > *N*_e_, where *N*_i_ denotes the average degree of polymerization of interdigitated segments and *N*_e_ is the critical degree of polymerization for entanglement formation.^51^ Figures 5 and 6 present a comprehensive summary of the modulus (Figure 5) and toughness (Figure 6) for all brush systems and their dependencies on the respective molecular parameters *M*_n_, *M*_w_, *Đ*, and ϕ_PMMA_ (i.e., the organic fraction). Note that values of the elastic modulus are normalized with respect to values of the linear reference polymer (hence *E*_N_, *U*_N_) to enable better comparison of values across materials and techniques. Figure 5 confirms that the (normalized) elastic modulus of low-dispersity (red symbols) and high-dispersity (blue symbols) systems followed similar trends. Particularly notable was the consistent trend of *E* with the organic content ϕ_PMMA_ across all systems (Figure 5d) as this confirmed that the modulus was dependent on the fraction of repeat units in the material, rather than their distribution among the different chains.

**Figure 5:**
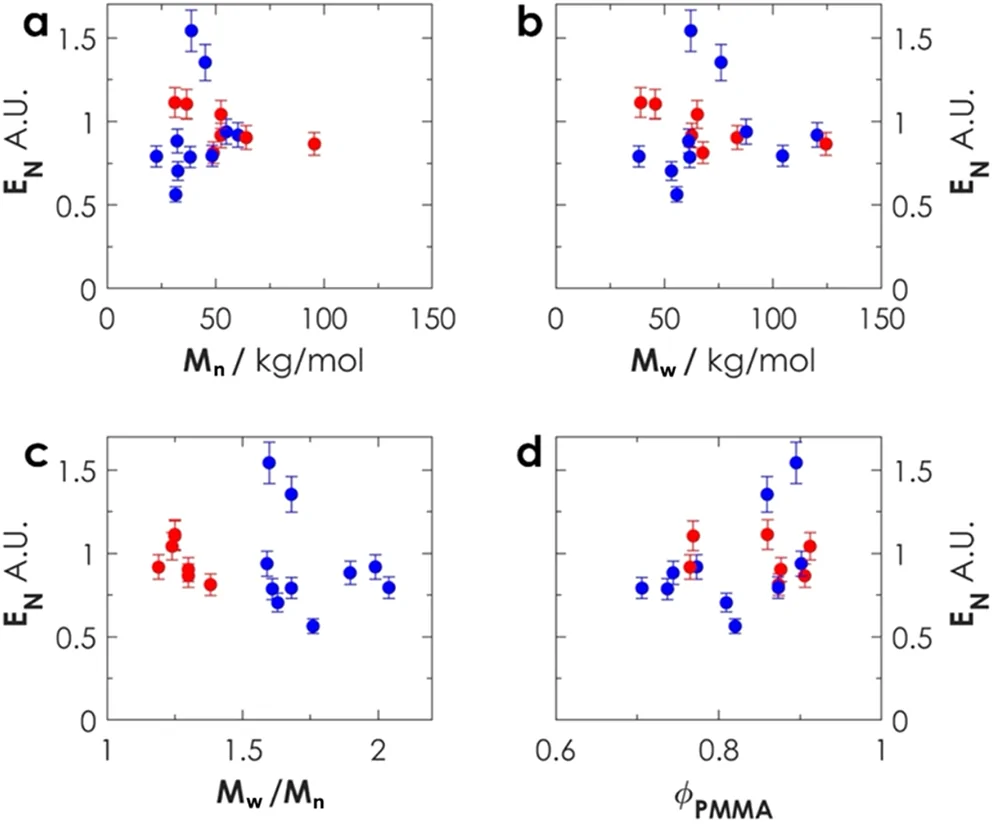
Normalized Young’s modulus (*E*_N_) of brush particle materials as functions of (a) *M*_n_, (b) *M*_w_, (c) *M*_w_/*M*_n_, and (d) ϕ_PMMA_. The modulus values are normalized with respect to the value of the linear PMMA reference. Red points are low dispersity; blue points are high dispersity. All brush particle sample moduli have been normalized by the measured modulus of *N* = 350 linear PMMA.

**Figure 6:**
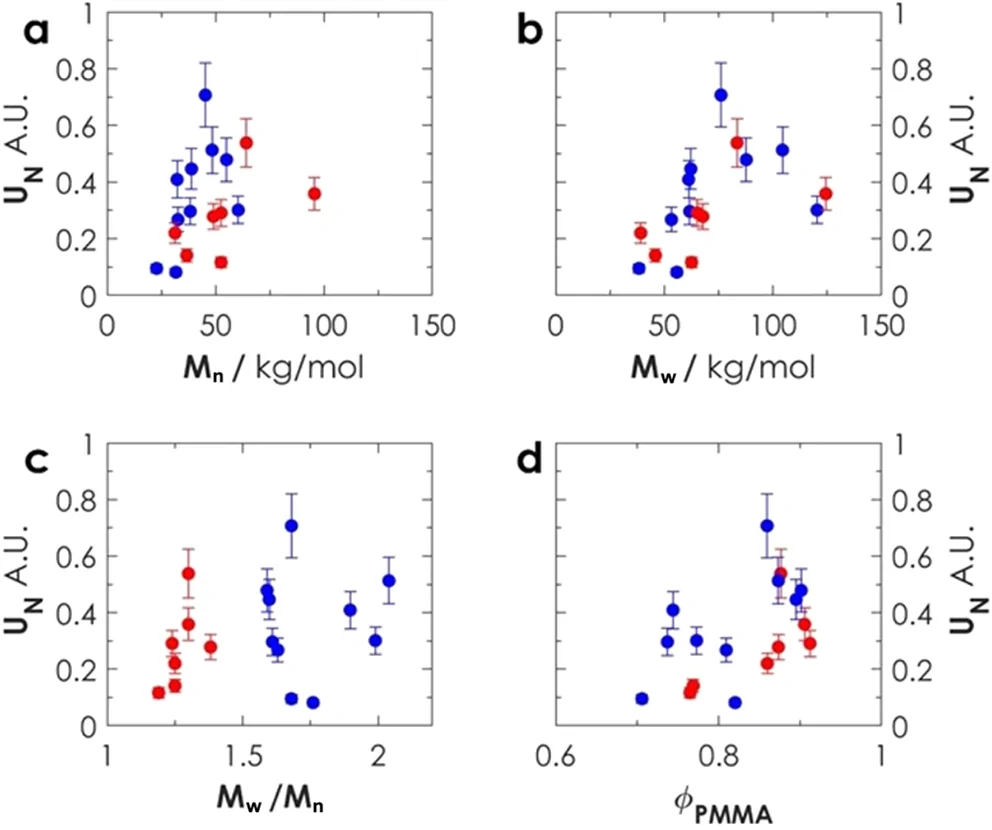
Normalized toughness (*U*_N_) of brush particle materials as functions of (a) *M*_n_, (b) *M*_w_, (c) brush dispersity *M*_w_ */M*_n_, and (d) organic content ϕ_PMMA_. Red points represent narrow dispersity systems; blue points high-dispersity systems. All brush particle sample toughness values have been normalized by the measured toughness of *N* = 350 linear PMMA.

Figure 6 reveals a more differentiated influence of brush dispersity on the toughness of brush particle films. For example, at given *M*_n_ the normalized toughness *U*_N_ of (most) disperse systems exceeds those of low-dispersity analogs (Figure 6a). This could be attributed to the contribution of the fraction of high-*M* chains to entanglement and craze formation in disperse systems. Accordingly, the trend is less pronounced when *M*_w_ is considered (Figure 6b) since the latter is sensitive to the high-molecular weight components in either system. Most notable is the effect of dispersity when the normalized toughness *U*_N_ is evaluated against ϕ_PMMA_. At a given fraction of organic component (i.e., equal number of repeat units in the system), Figure 6d reveals a consistently higher toughness for disperse brush materials. This implies that the distribution of repeat units in disperse brush architectures presents a more effective means to accomplish fracture-resistant brush particle films.

It is interesting to note that disperse samples consistently displayed a higher scatter in both *E* and *U* as compared to the narrow disperse analogues. Given the rather good reproducibility across the different material systems, this could indicate a minor influence of secondary parameters, such as microstructure, that possibly vary more inconsistently for dispersed brush particle systems.

Interestingly, the benefit of chain dispersity for the toughness of brush particle films decreased with increasing organic fraction. This is apparent from Figure 7, which displays the trend of fractional toughness of disperse and uniform brush particle solids with varying organic content (each point in Figure 7 represents a narrow and disperse brush particle pair with similar organic content, Figure S1). While in the limit of low organic content (ϕ_PMMA_ ∼ 0.7), the toughness of disperse systems exceeded that of uniform analogs by about 300%, the values converge at high organic content (ϕ_PMMA_ ∼ 0.9). Since all brush systems featured a comparable (low) grafting density, we expect this trend to reflect the influence of the average molecular weight as well as the asymmetry of the molecular weight distribution.

**Figure 7:**
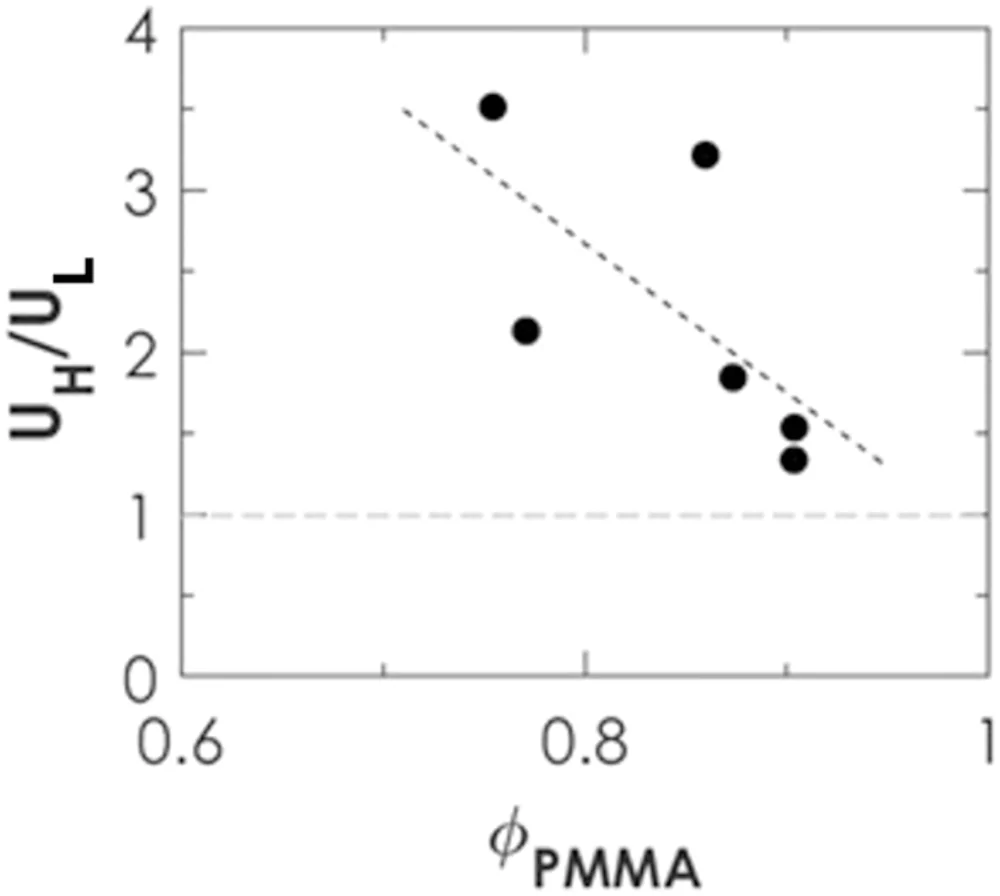
Ratio of normalized toughness of high- and low-dispersity brush particle solids at varying volume fraction of the organic component ϕ_PMMA_. The increase of toughness with decreasing ϕ_PMMA_ is attributed to the presence of high-molecular weight chains in disperse systems that promote entanglement formation at lower (number-) average molecular weights. The dotted line is to guide the eye. Particle pairs are detailed in Figure S1.

Toughness in glassy polymers has been shown to be related to microscopic flow processes (i.e., craze formation) during fracture.^52–54^ Crazing is contingent on the presence of entanglements, which can be expected to form if the degree of polymerization exceeds about twice the critical segment length (*N* > 2*N*_e_). In low-dispersity systems, the average degree of polymerization is a good estimator for the most frequent molecular weight of chains, and thus, the condition for entanglement formation is ⟨*N*⟩ = *N*_C_ = 2*N*_e_ ∼ 265 for PMMA. Comparison with the molecular characteristics of low-dispersity brush systems listed in Table 1 suggests that entanglement formation could be limited in systems L3 and L4 since ⟨*N*⟩ exceeds the entanglement limit by only a small amount. In contrast, disperse systems feature a “high-molecular weight tail” due to the positive skewness of MWDs (see asymmetry parameters *A*_s_ in Table 1; for the definition of *A*_s_, see the Supporting Information). This renders ⟨*N*⟩ a less consequential parameter for craze formation since the high molecular fraction of chains could contribute to entanglement formation even in systems for which the number-average molecular weight is below the entanglement limit. Recent studies by De Focatiis and co-workers^54^ examined the effect of MWD on craze formation for both bimodal blends and disperse polystyrenes. The authors reported that the presence of small amounts of high molecular fractions promoted craze formation and energy dissipation. To understand whether a similar mechanism was relevant for brush particle solids, the MWDs of all brush systems were determined from SEC traces after HF etching and subsequent conversion into frequency distributions of molecular weight (normalized to ∫*P*(*M*)­d*M* = 1). Figure 8 compares the frequency distributions for various low- and high-dispersity brush compositions, grouped by ϕ_PMMA_. For each pair in Figure 8, the percentage of chains above the defined crazing limit (*M*_C_) was calculated; the respective fractions are indicated as shaded areas.

**Figure 8:**
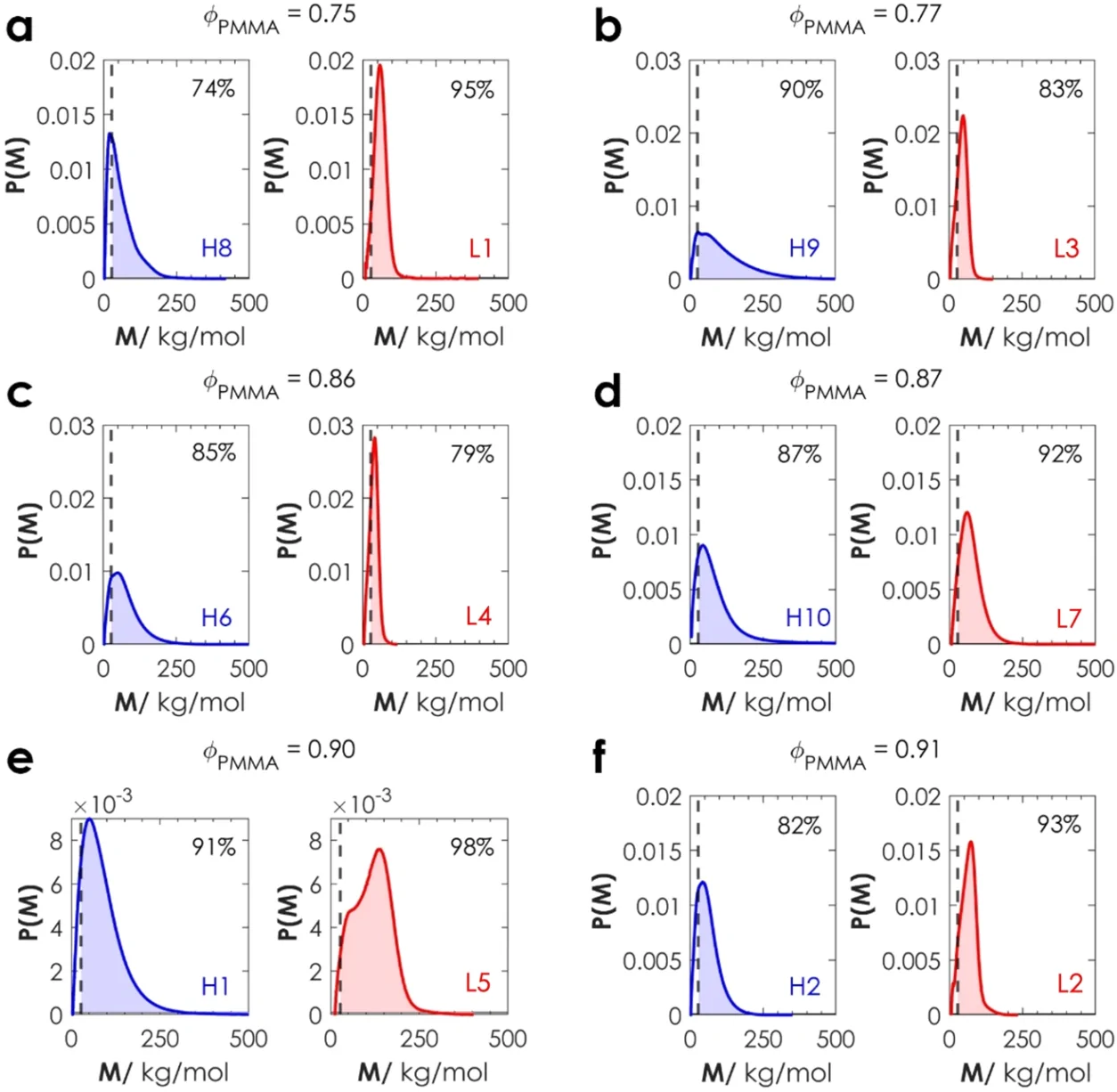
Distribution of chain molecular weights *P*(*M*) obtained from GPC after HF etching of silica cores. Frequency distributions were calculated using *P*(*M*_*i*_) = *s*_*i*_(*M*_*i*_)/*M*_*i*_, where *s*_*i*_(*M*_*i*_) denotes the (refractive index) detector signal for chains with molecular weight *M*_*i*_. Blue traces are high-dispersity samples; red traces are low-dispersity samples; and sample IDs are indicated in the bottom right corner of each trace. Shaded regions represent the fraction of chains of molecular weight greater than the crazing limit for PMMA, 2×*M*_*e*_ = 27.5 kg/mol,^51^ with the percentages displayed in the top right corner of each trace.

Several pertinent features can be observed from Figure 8. First, experimental MWDs of disperse systems are consistently positively skewed, in agreement with the expectation and prior reports of controlled radical polymerization processes.^40^ Therefore, the fraction of brush chains featuring a molecular weight greater than the threshold for craze formation was generally increased for disperse systems as compared to uniform brush systems; this trend notably diminished at higher organic fractions, consistent with the observed similarity of toughness values of high-molecular weight systems. To better understand the impact of dispersity, the “skewness (*U*_3_)” of distributions was thus determined by calculation of the third moment of a distribution about the mean. A widely used representation of the skewness *U*_3_ for polymer molecular weight distributions is the asymmetry coefficient *A*_s_ = *U*_3_/*s* ^3^, where *s* is the standard deviation of the distribution.^40^ For disperse brush particle systems, the asymmetry coefficients uniformly exceed those of the low-dispersity analogs (see Table 1). Note that even for systems with *M*_n_ ∼ *M*_C_ such as H8, a high asymmetry coefficient supported the hypothesis that the enhanced presence of very long chains above *M*_C_ contributed significantly to energy dissipation in strain to failure. Hence, the results suggest that the interdigitation between long chains in brush canopy layers increases with dispersity. The higher entanglement density promotes craze formation and hence increases the fracture toughness of disperse brush particle solids, even in the case of similar (number-) average molecular weight or a similar number of chains above the entanglement limit.

To validate the proposed increase of craze formation in high-dispersity brush particle films, the structure of cracks in brush particle monolayers was observed during the early stages of film rupture by electron imaging. In this process, brush particle multilayers were first cast on a poly­(acrylic acid) substrate and, after water immersion lift-off, transferred onto copper grids. Copper grids were bent over a surface with a curvature of 0.5 cm^–1^ to induce cracking. Figure 9 displays representative electron micrographs featuring cracks in narrow (L3, L5) and disperse (H1, H7) brush systems with a comparable organic fraction ϕ_PMMA_. Extensive craze formation is observed in both disperse brush compositions, while fracture surfaces in the respective uniform brush particle solids only show sporadic craze formation and brittle fracture at lower organic content. This supports the conclusion that in brush particle systems of comparable grafting density and organic content, craze formation is favored in more disperse brush compositions.

**Figure 9:**
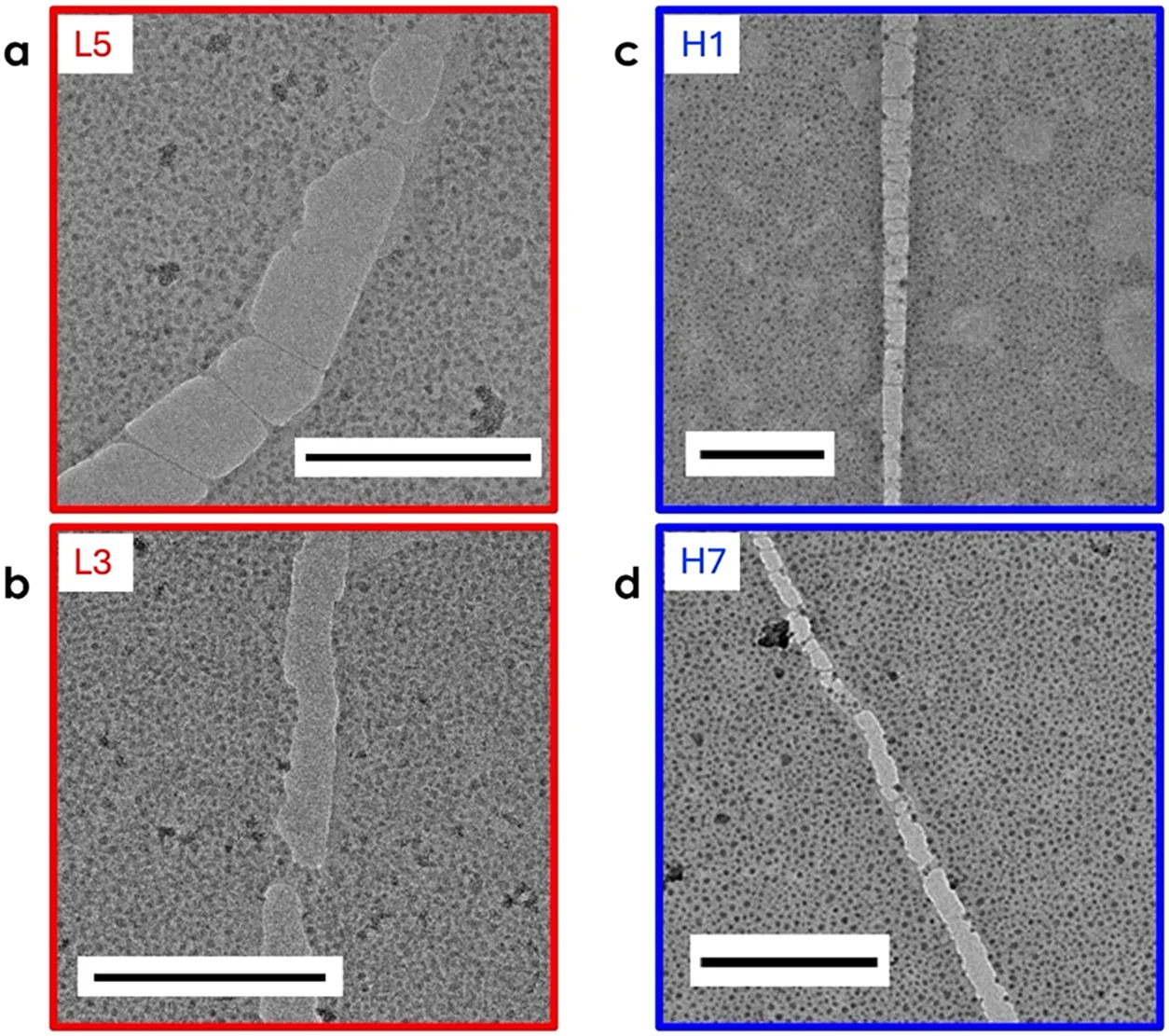
Bright-field TEM micrographs of crack surfaces during early fracture of brush particle materials (after bending deformation). Images have been contrast-enhanced to make polymer more visible. (a, b) Narrow dispersity samples L5 and L3, respectively; (c, d) high-dispersity samples H1 and H7, respectively. All scale bars represent 500 nm length.

## Conclusions

Our results demonstrate that disperse brush architectures enable a more effective ‘utilization’ of repeat units to promote mechanical robustness and, likely, processability of brush particle solids. For the tested systems, this benefit was realized without a detrimental impact on structural uniformity, as judged by the retention of uniformity in the Voronoi cell area distribution of monolayer films. The benefit of dispersity was most pronounced in the limit of low-molecular weight brush architectures as demonstrated by the negative correlation between toughness ratio of disperse and uniform (*H*:*L*) brush particle systems with organic content. This was rationalized by the positively skewed nature of brush MWDs in disperse systems, which promoted entanglement formation and crazing (and hence energy dissipation) during fracture even if the average brush degree of polymerization was below (or near) the entanglement limit. This suggests that the ‘engineering’ of MWDs (for example, via ARGET-ATRP) provides an alternative to bimodal brush architectures to realize mechanically robust brush particle solids with increased inorganic content. An interesting question for future research concerns the role of grafting density in the impact of brush dispersity. Since effective craze formation requires the interdigitation of brush layers between adjacent particles, the impact of brush dispersity could be altered in the limit of high grafting density (i.e., dense brush architectures).

## Supplementary Material



## Notes

Electron micrographs of all samples along with average Voronoi cell area distribution parameters, thermal history of samples and determination of the glass transition temperature, and calculation of skewness and the asymmetry coefficient (PDF)

## §

Intel Corporation, 2501 NE Century Boulevard, Hillsboro, Oregon 97124, United States

## ∥

School of Physical Science and Technology, ShanghaiTech University, Shanghai 201210, China.
