# Supplementary material for: Mechanical Properties of Glassy Brush Particle Solids Featuring Chain Dispersity
Source: Langmuir. 2026 Sep 1;42(36):26338–46. doi: 10.1021/acs.langmuir.6c02933 (PMC13585126; doi:10.1021/acs.langmuir.6c02933)
Supplement: Supplementary file 1 [file la6c02933_si_001.pdf]

Supporting Information

Mechanical Properties of Glassy Brush Particle Solids Featuring Chain Dispersity

Ayesha Abdullah[1], Hanshu Wu[2], Yuqi Zhao[1], Jaejun Lee[1†], Jiajun Yan[2‡], Krzysztof Matyjaszewski[2], Michael R. Bockstaller[1]*

[1] Department of Materials Science and Engineering, Carnegie Mellon University, Pittsburgh, Pennsylvania, 15213, United States.

[2] Chemistry Department, Carnegie Mellon University, Pittsburgh, Pennsylvania, 15213, United States.

* to whom correspondence should be addressed: bockstaller@cmu.edu

† current address: Intel Corporation, 2501 NE Century Blvd, Hillsboro, OR 97124, United States

‡ current address: School of Physical Science and Technology, ShanghaiTech University, Shanghai 201210, China

Microstructure and Voronoi Cell Analysis Using TEM Micrographs

Quantitative analysis of TEM micrographs of brush particle material was performed through Voronoi cell analysis on particle centers using MATLAB. Figure S1 shows representative TEM micrographs (unprocessed) of samples N6, H10, and N4. Image processing, exemplified for sample N6 shown in Figure S1, was performed as follows: Gaussian blurring to reduce noise for better recognition; erosion and dilation to separate neighboring particles and reduce high-contrast impurities; adaptive thresholding with neighborhood size on the length scale of the size of the particle in the image; binarization (distinct features include the bright polymer background and dark silica particles) using the threshold; particle recognition and filtering by size (to remove anomalous spots); and Voronoi tessellation of the particle centroids. The results of each step are shown in Figure S2.

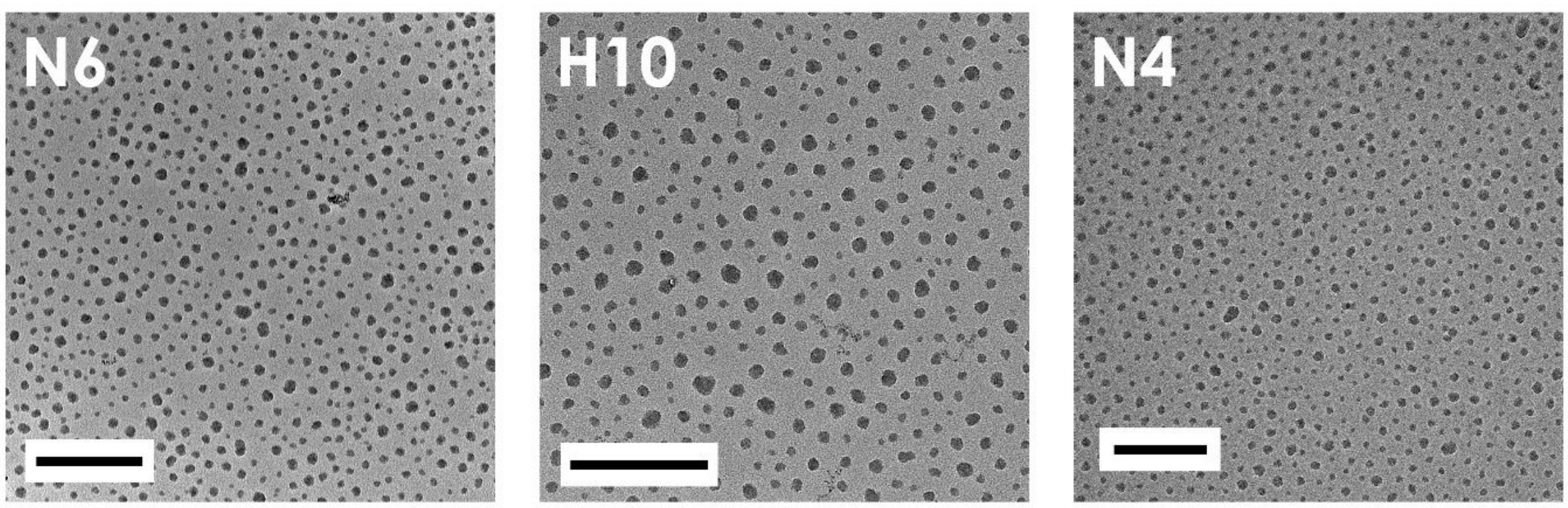

Figure S1. Example TEM micrographs collected of PMMA-SiO_2 monolayers for samples (left to right) N6, H10, N4; sample IDs correspond to those in the text. The light background is the nearly electron transparent PMMA, while the darker particles are highly scattering amorphous silica, enabling binarization. All scale bars are 200nm.

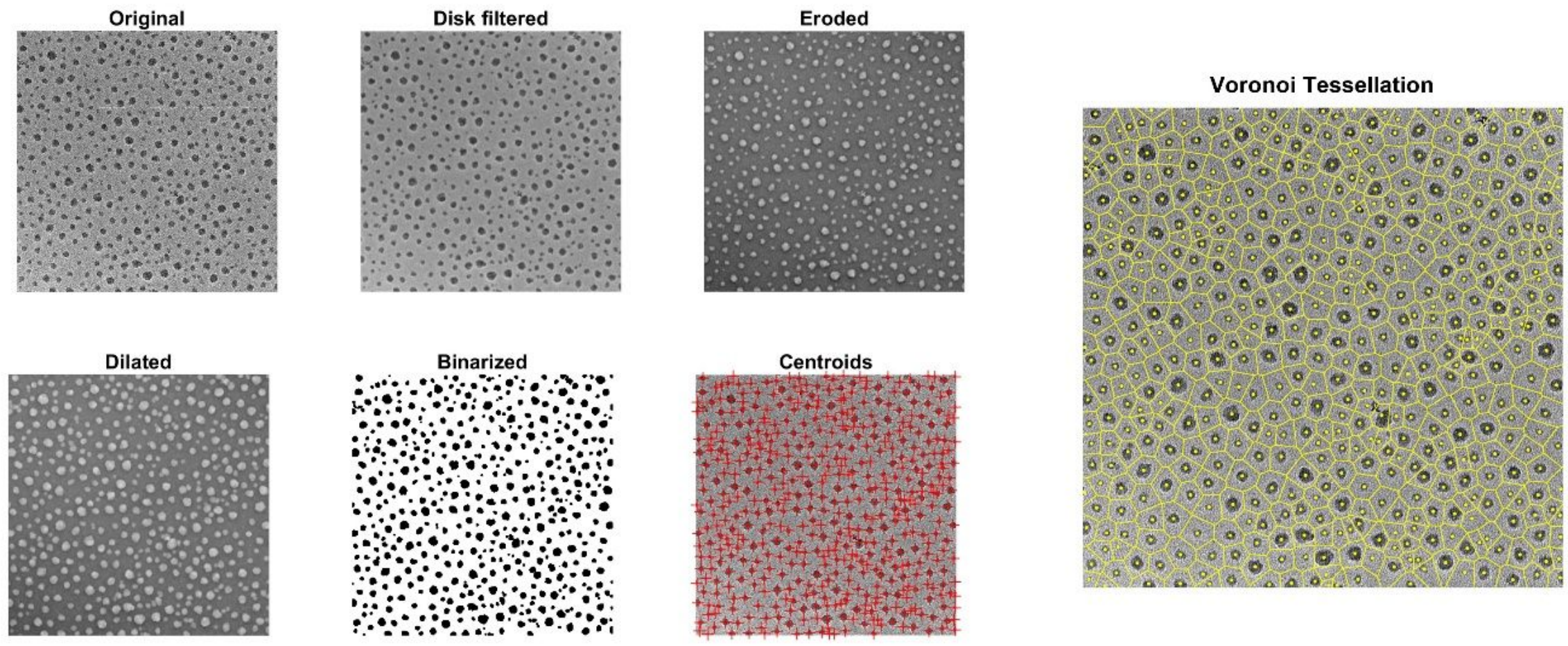

Figure S2. Representative image processing of a TEM micrograph for sample N6 including filtering, erosion/dilation, binarization using adaptive thresholding, centroid-picking of particle domains, and resultant Voronoi tessellation of particle centroids.

The resultant Voronoi cell area distribution is shown in Figure S3; cells lying on the edge of the image are excluded from this analysis to reduce inaccurate edge effects.

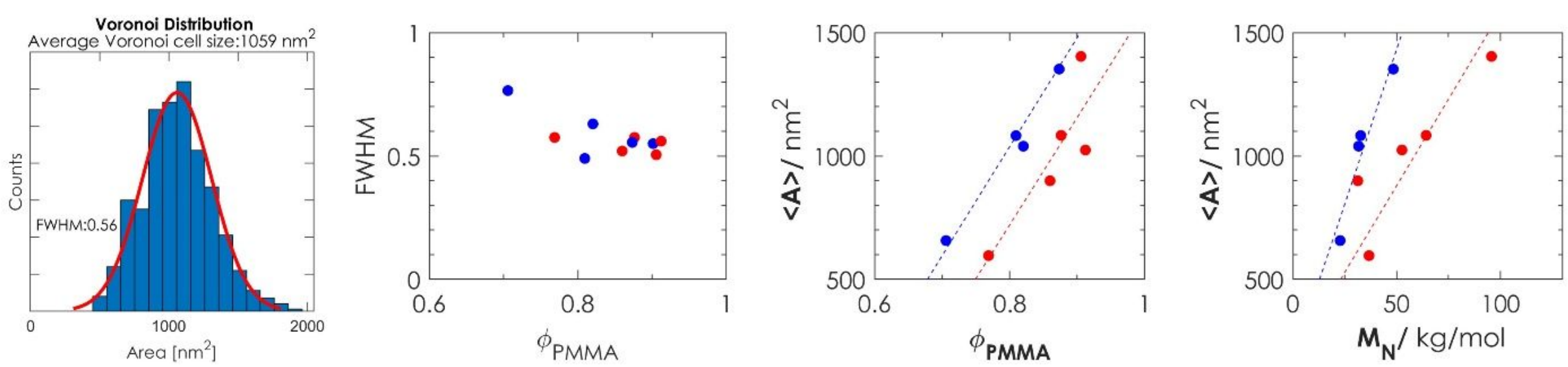

Figure S3. Left to right: 1) Voronoi cell area distribution of sample N6 resulting from the analysis depicted in Figure S2; the average cell area size and FWHM are indicated. 2) FWHM of high dispersity (blue) and narrow dispersity (red) samples over organic fraction, revealing consistent uniformity. Average Voronoi cell area size over (3) organic fraction ϕ_{PMMA}, and (4) M_N. Linear fits are provided to guide the eye to the positive relationships in panels 3 and 4.

Differential Scanning Calorimetry and Determination of Glass Transition

Figure S4 shows representative thermal history of narrow (red) and high (blue) dispersity brush particle samples N5 and H1 respectively. The glass transition temperature T_g, marked by a dotted line in Figure S4, was determined using the inflection point of the traces, calculated at the minimum of the derivative of heat flow.

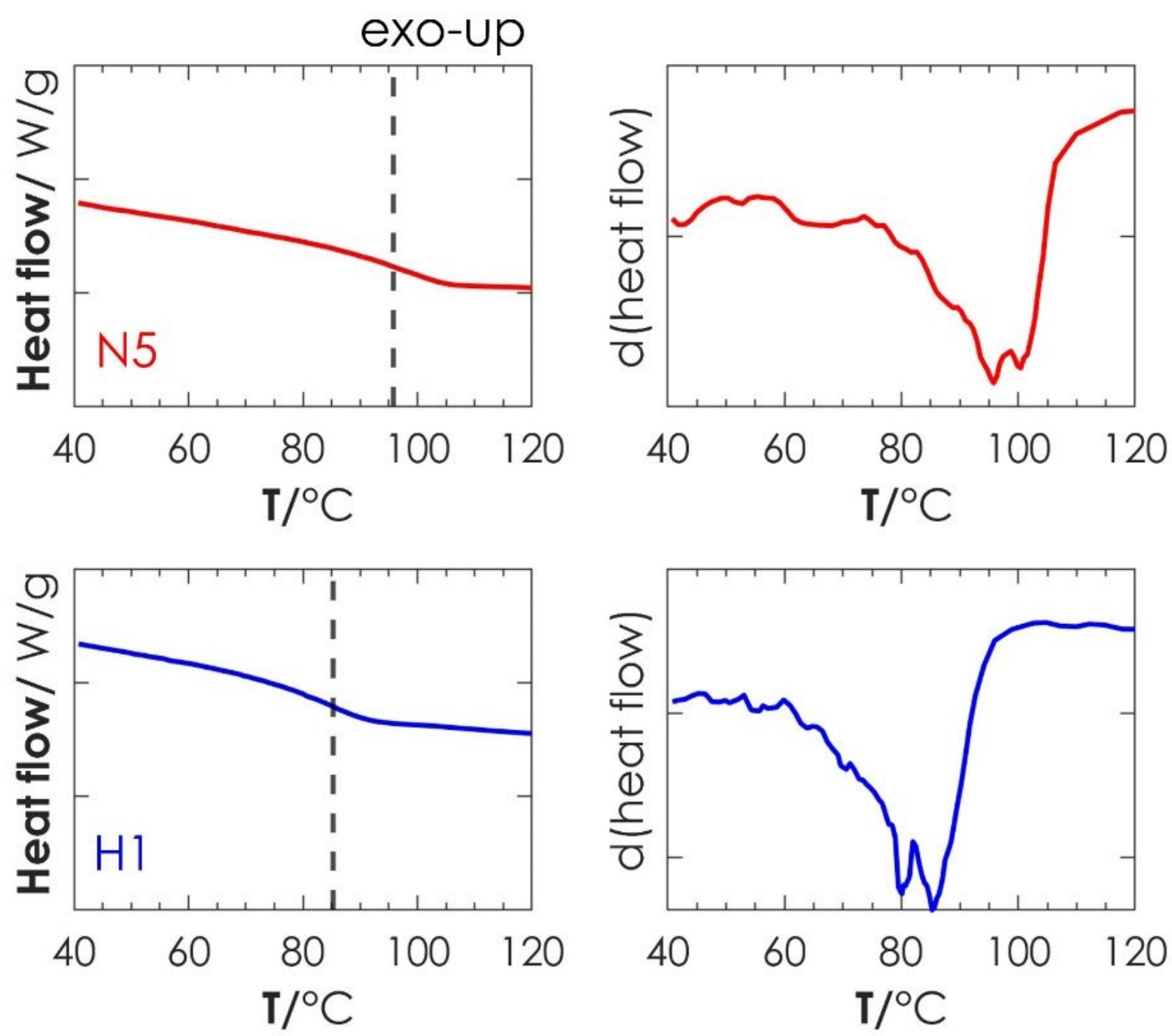

Figure S4. Representative thermal history (left) for narrow (red) and high (blue) dispersity brush particle samples N5 and H1. The glass transition temperature T_g is indicated by the dotted line, and was determined by finding the inflection point; this is the minimum of the derivative of the heat flow (right) for each respective sample.

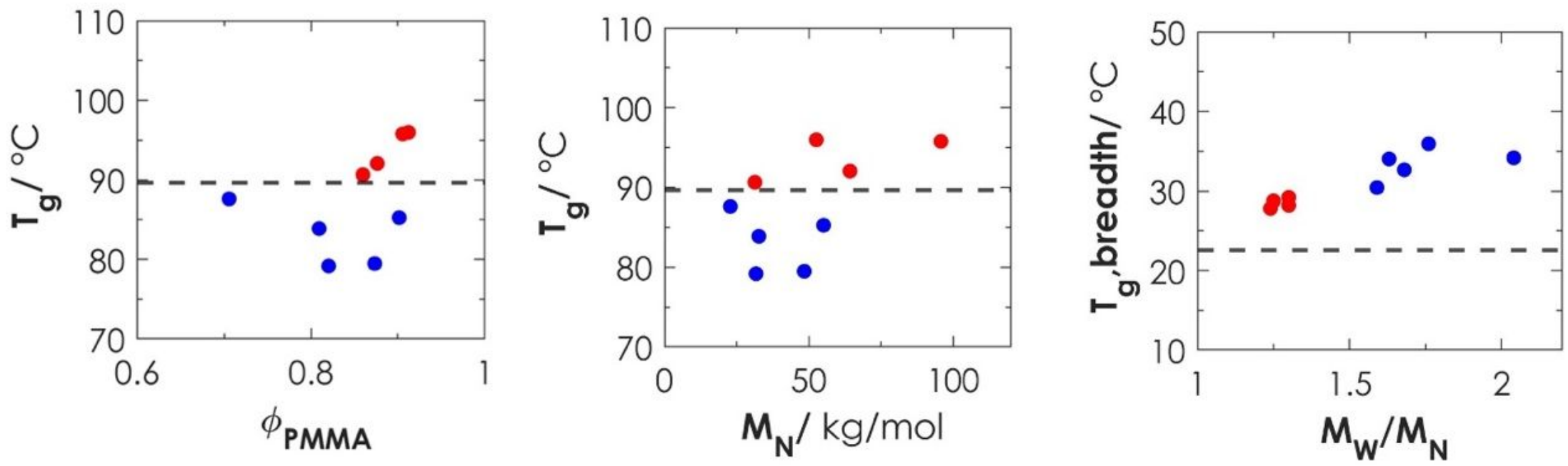

Figure S5. Left to right: Glass transition temperature T_g of high (blue) and narrow (red) dispersity brush particle materials over ϕ_{PMMA} (1) and M_N (2). 3) Breadth of the glass transition over dispersity, or M_W/M_N, of brush particle materials. The dotted line indicates the respective property measured for N = 337 linear PMMA as a reference.

The summary of molecular characteristics and thermal transition temperatures of all systems is provided in Table S1.

Table S1. Molecular characteristics of brush particle systems.

Sample ID	ϕ_{PMMA}	N	M_N (g/mol)	M_W (g/mol)	σ (nm^{-2})	$Đ$	A_S	T_g (°C)	T_g breadth
Linear PMMA	1.00	350	35,000	38,500	–	1.10	–	89.6	24.6
N1	0.76	526	52,550	62,535	0.11	1.19	1.50	-	-
N2	0.91	526	52,570	65,187	0.35	1.24	0.42	96.0	27.8
N3	0.77	367	36,690	45,863	0.16	1.25	0.34	-	-
N4	0.86	313	31,300	39,125	0.35	1.25	0.30	90.7	28.8
N5	0.91	957	95,690	124,397	0.18	1.30	0.58	95.8	29.2
N6	0.88	642	64,200	83,460	0.20	1.30	0.33	92.0	28.2
N7	0.87	490	49,000	67,700	0.25	1.38	1.16	-	-
H1	0.90	551	55,070	87,740	0.30	1.59	1.94	85.3	30.4
H2	0.90	389	38,910	62,180	0.39	1.60	1.43	-	-
H3	0.74	383	38,320	61,695	0.13	1.61	1.36	-	-
H4	0.81	327	32,650	53,250	0.23	1.63	1.82	83.9	34.0
H5	0.71	228	22,790	38,300	0.19	1.68	1.87	87.6	32.7
H6	0.86	453	45,290	76,087	0.24	1.68	1.67	-	-
H7	0.82	317	31,680	55,730	0.26	1.76	1.89	79.2	35.9
H8	0.74	323	32,260	61,190	0.16	1.90	2.26	-	-
H9	0.77	605	60,480	120,355	0.10	1.99	2.25	-	-
H10	0.87	484	48,400	104,400	0.25	2.04	5.20	79.5	34.2

Hysteretic Elastic Behavior of PMMA and Brush Particle Materials

To confirm the elastic strain limit used to determine Young's modulus *E* of linear PMMA and PMMA-SiO_2 brush particle materials, defined in the text as 0.003 mm/mm, free standing linear polymer and brush particle films were tested. The materials and specimen dimensions (presented as $L \times w \times t$) are noted in Figure S6. The samples were strained to 0.003 mm/mm at the identical rate of the tensile test (0.01 mm/s), returned to their original length, and then strained again, leading to two tensile straining phases, and two 'relaxation' phases where the original length was recovered. Figure S6 exhibits negligible hysteresis in both linear polymer and brush particle material, with both samples nearly perfectly recovering their original state. Additionally, the Young's modulus measured in each tensile phase exhibited less than 3% deviation, thus confirming linearly elastic behavior in this strain limit.

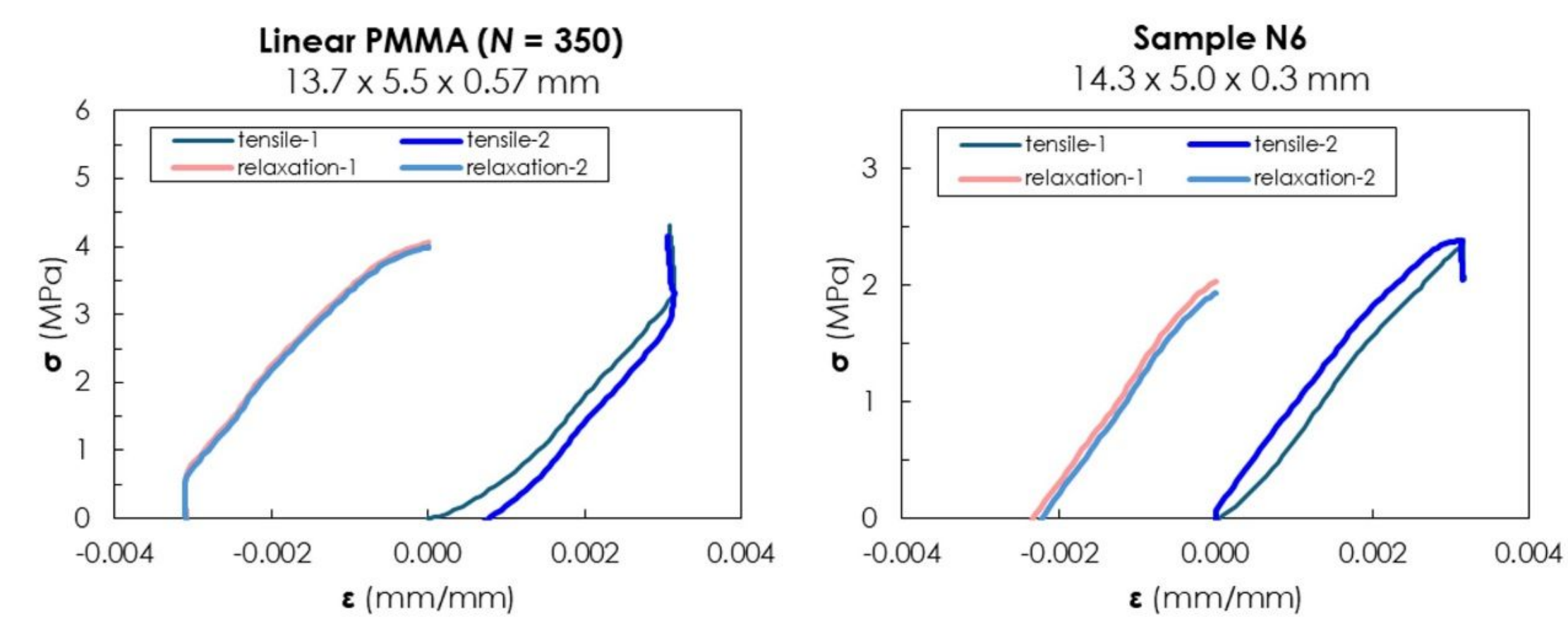

Figure S6. Tensile and recovery curves obtained during an oscillatory tension experiment to observe hysteresis (or lack thereof) in linear PMMA (left) and brush particle sample N6 (right) free standing films. The specimen dimensions are indicated in the figure.

Calculation of MWD Skewness and the Asymmetry Coefficient, A_s

To evaluate the asymmetry of MWDs of disperse brush systems, the skewness U_3 was determined from the third moment of the distribution about the *mean* following to

$$U_3 = \frac{\sum n_i (M_i - M_N)^3}{\sum n_i} \tag{S3}$$

Where n_i is the frequency of chains with molecular weight M_i. In addition, the asymmetry coefficient A_s was determined as alternate descriptor for the 'tailed' character of disperse MWDs.

$$A_S = \frac{U_3}{s^3} \tag{S4}$$

Where s is the standard deviation of the MWD.

Dependence of Relative Toughness on Organic Content and Dispersity

To evaluate the dependence of U_L/U_H on the polymer content, brush particle samples were paired according to similar organic content. Table S2 summarizes the pairs.

Sample ID	ϕ_{PMMA}	N	M_n (g/mol)	M_w (g/mol)	σ (nm^{-2})	$Đ$	A_S
Linear PMMA	1.00	350	35,000	38,500	–	1.10	–
H8	0.74	323	32,260	61,190	0.16	1.90	2.26
L1	0.76	526	52,550	62,535	0.11	1.19	1.50
H9	0.77	605	60,480	120,355	0.10	1.99	2.25
L3	0.77	367	36,690	45,863	0.16	1.25	0.34
H6	0.86	453	45,290	76,087	0.24	1.68	1.67
L4	0.86	313	31,300	39,125	0.35	1.25	0.30
H10	0.87	484	48,400	104,400	0.25	2.04	5.20
L7	0.87	490	49,000	67,700	0.25	1.38	1.16
H1	0.90	551	55,070	87,740	0.30	1.59	1.94
L5	0.91	957	95,690	124,397	0.18	1.30	0.58
H2	0.90	389	38,910	62,180	0.39	1.60	1.43
L2	0.91	526	52,570	65,187	0.35	1.24	0.42
